# Characteristics of the Top 50 Most‐Cited Arthroscopic Remplissage Articles Reveal Growing Recognition But Persistent Knowledge Gaps

**DOI:** 10.1002/ars2.70001

**Published:** 2026-09-25

**Authors:** Brian S. Tao, Ross Clarke, Kevin A. Hao, Antonio Cusano, John D. Kelly, Robert L. Parisien, Richard Ma, Xinning Li

**Affiliations:** ^1^ Boston University School of Medicine Boston Massachusetts U.S.A.; ^2^ Department of Orthopaedic Surgery & Sports Medicine University of Florida Gainesville Florida U.S.A.; ^3^ Department of Orthopaedic Surgery University of Connecticut Farmington Connecticut U.S.A.; ^4^ Department of Orthopaedics University of Pennsylvania Philadelphia Pennsylvania U.S.A.; ^5^ Department of Orthopaedics Icahn School of Medicine at Mount Sinai New York New York U.S.A.; ^6^ Department of Orthopaedic Surgery University of Missouri School of Medicine Columbia Missouri U.S.A.

## Abstract

**Purpose:**

To identify and examine the article, author, and journal characteristics of the top 50 most‐cited articles on the arthroscopic remplissage procedure for the treatment of anterior shoulder instability in the field of orthopaedic sports medicine.

**Methods:**

A bibliometric analysis was performed for the most‐cited arthroscopic remplissage articles using the Web of Science databases with the search term “remplissage” OR “posterior capsulotenodesis” OR “infraspinatus tenodesis.” Articles were included if their primary focus was on the arthroscopic remplissage technique and with or without associated clinical outcomes. Articles addressing shoulder instability, shoulder dislocation, and surgical decision making without explicit reference to the arthroscopic remplissage technique were excluded.

**Results:**

The top 50 articles on arthroscopic remplissage received a total of 2727 citations with an average of 55 citations per year. Citations have increased from 2008 to 2024. The most frequently cited article with the highest citations‐per‐year was Purchase et al. (2008), which introduced the arthroscopic remplissage technique. The top three articles were cited >100 times. The top‐cited articles were predominantly published in *Arthroscopy*, authored most frequently (first or co‐author) by Grant Garcia (10%), with the majority originating from the USA (38%), especially from University of Pennsylvania (8%). No relationship was found between level of evidence and number of citations (*P* = .69). Adjusted for study design and level of evidence, there was a 1.3 increase in citations per year for every 1 unit increase in journal impact factor (95% CI: 0.28‐2.38, *P* = .02).

**Conclusions:**

No relationship was found between the level of evidence and number of citations of arthroscopic Remplissage articles; however, journal impact factor was associated with citation frequency when adjusted for study design and level of evidence.

**Clinical Relevance:**

Articles with the most citations often correlate with greater influence by shaping clinical decision‐making and guiding future research. By identifying and characterizing the recurrent themes of the top‐cited articles, we provide a comprehensive overview of the evolution, current understanding, and impact of the arthroscopic remplissage technique in the treatment of anterior shoulder instability.

## Graphical abstract

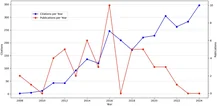

Among patients with anterior shoulder instability, the presence of a Hill‐Sachs lesion—a posterolateral humeral head defect—has been identified as a potential factor that may increase recurrence rates, particularly when the defect is “off‐track” and engages with the glenoid rim.^1^ Historically, open capsular shift was regarded as the gold‐standard treatment for anterior shoulder instability. With advancements in arthroscopic techniques, arthroscopic Bankart repair (ABR) has now become the cornerstone of surgical treatment in patients with minimal glenoid bone loss.^2^, ^3^ However, its limitations in addressing significant Hill‐Sachs lesions in addition to the higher failure rates in patients with anterior glenoid bone loss (>critical value) have led to an exploration of other adjunctive procedures.^4^, ^5^

Remplissage (French for “to fill in”) is an arthroscopic technique that fills in the Hill‐Sachs defect by securing the posterior capsule and infraspinatus tendon into it, rendering the lesion extra‐articular and preventing engagement with the anterior glenoid rim. Eugene Wolf described the remplissage technique and performed the operation as early as 2002.^6^, ^7^ Purchase et al.^8^ in 2008 described the arthroscopic remplissage technique, a capsulotenodesis procedure that fills the Hill‐Sachs defect with the infraspinatus tendon and posterior capsule to prevent anterior engagement of the HS defect with the glenoid rim. Supporting its use, Boileau et al.^9^ (2012) evaluated the anatomical and functional outcomes of ABR with remplissage in 47 patients with large engaging Hill‐Sachs lesions and no glenoid bone loss. At a mean follow‐up of 24 months, 98% of patients showed shoulder stability, with a high rate of return to play (90%) and return to sport‐specific activity (68%). Although 100% of cases showed healing of the posterior capsule and infraspinatus into the humeral defect, approximately 10° of external rotation (ER) loss was observed, underscoring a trade‐off between enhanced stability and range of motion limitations. Additionally Frantz et al.^10^ found 26% of patients had ≥20° ER deficit with the arm at the side, 42% had similar deficits when the arm was abducted. The pros and cons of remplissage remain unclear, as the decision involves more than simply balancing potential loss of ER against anterior shoulder instability.^11^, ^12^, ^13^ Despite potential concerns regarding potential postoperative range‐of‐motion limitations in ER due to the tenodesis effect, the remplissage procedure has grown in appreciation due to its protective effect in decreasing failure rate after ABR and is increasingly used in cases of recurrent instability with concomitant Hill‐Sachs lesions both “on” or “off” track.^14^ Additionally, remplissage has shown to be protective for “near‐track” lesions, which are Hill‐Sachs defects technically classified as ‘on‐track’ but lie within 10 mm of the glenoid track margin, thereby placing them at higher risk for engagement and instability.^15^

Bibliometric studies provide valuable insights into the evolution of research and its impact on clinical practice. Although similar bibliometric analyses have been performed in other areas of orthopaedics and shoulder surgery, little is known about how it pertains to the arthroscopic remplissage procedure.^16^, ^17^, ^18^ The purpose of this study was to identify and examine the article, author, and journal characteristics of the top 50 most‐cited articles on the arthroscopic remplissage procedure for the treatment of anterior shoulder instability in the field of orthopaedic sports medicine. We hypothesized that no relationship will be found between level of evidence and the number of citations and that the manuscript by Purchase et al.^8^ will be the most‐cited article.

## METHODS

All databases in Web of Science, which includes Web of Science Core Collection, Chinese Science Citation Database, Russian Science Citation Index, Arabic Citation Index, and Book Citation Index, and Scientific Electronic Library Online, was queried on February 3, 2025 using the following Boolean search term: “remplissage” OR “posterior capsulotenodesis” OR “Infraspinatus tenodesis.” Web of Science was chosen due to the amount of articles indexed, along with the use of multiple international databases (Arabic Citation Index, Russian, Chinese) beyond its core collection. All articles since inception were considered. To improve sensitivity, no filters were added. Articles were included if the primary focus was related to the remplissage technique. Articles addressing shoulder instability, shoulder dislocation, and surgical decision making without explicit reference to remplissage were excluded.

The top 50 most‐cited articles were reviewed by the first two authors (B.T., R.C.) to obtain article information including author names, publication year, country of origin, journal name, total number of citations, and the level of evidence. If there was no consensus on inclusion/exclusion of an article or information extracted from an article, it was left to the discretion of the senior author (X.L.). Inter‐rater reliability between the two reviewers was quantified via Cohen's k statistic with Landis & Koch thresholds: <0 is poor, 0.00 to 0.20 slight, 0.21 to 0.40 fair, 0.41 to 0.60 moderate, 0.61 to 0.80 substantial, and 0.81 to 1.00 almost perfect agreement.

Analysis was performed in Python 3.10.0 (Python Software Foundation, Wilmington, DE, USA), and plots were generated using the Matplotlib 3.8.4 and Plotly 5.23.0 (Plotly Technologies, Montreal, QC). Network analysis was performed using the NetworkX 3.4.1 package with article keywords when available. In bibliometric network analysis, a high degree of centrality indicates that a keyword is strongly connected to many others, reflecting its prominence and influence in shaping key themes across the most‐cited remplissage articles. Spearman coefficient analysis was used to assess the correlation of a study's level of evidence and the amount of citations received. Multivariable linear regression adjusted for study design and level of evidence was used to identify potential relationship of journal impact factor on an article's citations per year (CPY). A sensitivity analysis was also conducted by excluding data points exceeding two standard deviations from the mean. Calculations of citation per year (number of citations/years since publication) used 2024 as the most recent year for analysis. Analysis was also performed on citation density (the number of citations for an article based on the year of publication). CPY was calculated as total citations divided by years since publication (through 2024), whereas citation density emphasized citations relative to the publication year. This distinction influenced article rankings, with CPY favoring consistently cited older articles and density highlighting newer articles with rapid early impact. Country of origin was determined by the affiliation of the first author for each article. If level of evidence was not explicitly reported for an article, *The Journal of Bone and Joint Surgery* guidelines were used.^19^ Impact factor for each journal was gathered from the official Journal Citation Reports (Clarivate PLC, London, UK), based on the most recent year of data available at the time of access.^20^

## RESULTS

The top 50 articles received 2727 citations with an average of 55 ± 41 citations per article and a range of 22 to 266 citations (Supporting Information 1). In terms of CPY, the top fifty articles had an average of 6 ± 3 CPY, with a minimum of 2.2 CPY and a maximum of 15.6 CPY. Of note, the top three articles were each cited more than 100 times. Inter‐rater reliability with Cohen k was 0.65, indicating substantial agreement between reviewers.

### Characteristics of the Top Ten Most‐Cited Remplissage Articles

The most‐cited remplissage article (n = 266) is the technical article detailing the remplissage procedure and was published in *Arthroscopy* in 2008 by Purchase et al.^8^ This article details how to prevent engagement with the glenoid by converting a Hill‐Sachs lesion from an intra‐articular defect into an extra‐articular one by “filling it in” with the infraspinatus tendon and posterior capsule or the “Remplissage” procedure. The second most‐cited article (n = 156) was Boileau et al.,^9^ published in *The* *Journal of Bone and Joint Surgery – American Volume* in 2012. This comparative study evaluated the remplissage procedure for large, engaging Hill‐Sachs lesions (Calandra grade III) without substantial glenoid bone loss. The authors reported promising outcomes, including high rates of return to sports and shoulder stability. The third most‐cited article (n = 114) was published in *Arthroscopy* in 2009 by Koo et al.^21^ This technical article describes a modified remplissage technique where the anterior Bankart repair sutures are placed first but not tied until after the humeral suture anchors have been placed in the Hill‐Sachs lesion and the subacromial bursa is cleared before placing two transtendon suture anchors in the Hill‐Sachs lesion. The research topic and results of the top ten most‐cited remplissage articles are presented in Table 1. The top ten remplissage articles by CPY is reported in Table 2.

**TABLE 1:** Top Ten Remplissage Articles by Citations

Rank	Title	First Author	Summary
1	Hill‐Sachs remplissage: An arthroscopic solution for the engaging Hill‐Sachs lesion^8^	Purchase, Robert J.	Technique note for remplissage describing the process of converting a Hill‐Sachs lesion from an intra‐articular defect to an extra‐articular one by filling it with the infraspinatus tendon and posterior capsule, preventing engagement with the glenoid. An extension of Wolf et al.'s work in 2004
2	Anatomical and functional results after arthroscopic Hill‐Sachs remplissage^9^	Boileau, Pascal	Remplissage used for 47 patients with large Hill‐Sachs lesion (Calandra grade III), engaging over the glenoid rim, without substantial glenoid bone loss. Results showed return to sports rate of 90% and stable shoulder rate of 97% in patients
3	Arthroscopic double‐pulley remplissage technique for engaging Hill‐Sachs lesions in anterior shoulder instability repairs^21^	Koo, Samuel S.	Technique note for modified remplissage where the anterior Bankart repair sutures are placed first but not tied until after the humeral suture anchors have been placed in the Hill‐Sachs lesion, and the subacromial bursa is cleared before placing two transtendon suture anchors in the Hill‐Sachs lesion
4	Arthroscopic Bankart repair combined with remplissage technique for the treatment of anterior shoulder instability with engaging Hill‐Sachs lesion a report of 49 cases with a minimum 2‐year follow‐up^22^	Zhu, Yi‐Ming	Case series of 49 patients showing remplissage able to restore shoulder stability without significant impairment of shoulder function. Results of active forward elevation improvement of 8°, loss of 1.9° of external rotation to the side, improvements in ASES score, Constant score, Rowe score. Successful healing of infraspinatus tendon and failure rate of 8.2%
5	Outcomes of the remplissage procedure and its effects on return to sports average 5‐year follow‐up^23^	Garcia, Grant H	Case series of 50 patients looking at long term outcomes of remplissage at average 60.7 months follow‐up. Redislocation rate after remplissage was 11.8% with 95.5% of patients returning to full sports at an average of 7 months
6	Remplissage versus modified Latarjet for off‐track Hill‐Sachs lesions with subcritical glenoid bone loss^24^	Yang, Justin Shu	Comparing remplissage (n = 98) and modified Latarjet (n = 91) procedures, remplissage had higher VAS score, higher recurrence rate, higher revision rate, and lower complication rate
7	Hill‐Sachs remplissage, an arthroscopic solution for the engaging Hill‐Sachs lesion: 2‐to 10‐year follow‐up and incidence of recurrence^6^	Wolf, Eugene M	Case series of 45 patients treated with remplissage at average 58 mo follow‐up. Results showed great Rowe score, Constant score, and Western Ontario Shoulder Instability Index. No reoperations or complications were reported
8	A prospective, comparative, radiological, and clinical study of the influence of the remplissage procedure on shoulder range of motion after stabilization by arthroscopic Bankart repair^11^	Nourissat, Geoffroy	Prospective study at 1 and 2 year follow‐up of bankart vs bankart with remplissage showing no difference in range of motion. However, 1/3 of remplissage patients had posterosuperior pain
9	Arthroscopic Hill‐Sachs remplissage a systematic review^25^	Buza, John A., III	Systematic review of 6 studies on remplissage outcomes. Results showed good postoperative clinical outcome scores, no significant loss of shoulder motion, favorable failure rate, and low overall complication rates
10	Remplissage repair‐new frontiers in the prevention of recurrent shoulder instability a 2‐year follow‐up comparative study^26^	Franceschi, Francesco	Two groups of 25 patients who underwent either Bankart or Bankart with remplissage and minimum of 2 year follow‐up. Significant postoperative improvements with no significant differences between the groups for active forward elevation, external rotation besides the body, internal rotation, UCLA score, Constant score, and Rowe score

UCLA, University of California Los Angeles; VAS, visual analog scale.

**TABLE 2:** Top Ten Most‐Cited Remplissage Articles by Citations Per Year

Rank	Title	Authors	Citations Per Year	Total Citations
1	Hill‐Sachs remplissage: An arthroscopic solution for the engaging Hill‐Sachs lesion^8^	Purchase, Robert J.; Wolf, Eugene M.; Hobgood, E. Rhett; Pollock, Michael E.; Smalley, Chad C.	15.6	266
6	Remplissage versus modified Latarjet for off‐track Hill‐Sachs lesions with subcritical glenoid bone loss^24^	Yang, Justin Shu; Mehran, Nima; Mazzocca, Augustus D.; Pearl, Michael L.; Chen, Vincent W.; Arciero, Robert A.	13.1	92
19	Arthroscopic Bankart repair with and without arthroscopic infraspinatus remplissage in anterior shoulder instability with a Hill‐Sachs defect: A randomized controlled trial^27^	MacDonald, Peter; McRae, Sheila; Old, Jason; Marsh, Jonathan; Dubberley, Jamie; Stranges, Greg; Koenig, James; Leiter, Jeff; Mascarenhas, Randy; Prabhakar, Sharad; Sasyniuk, Treny; Lapner, Peter	13	52
2	Anatomical and functional results after arthroscopic Hill‐Sachs remplissage^9^	Boileau, Pascal; O’Shea, Kieran; Vargas, Pablo; Pinedo, Miguel; Old, Jason; Zumstein, Matthias	12	156
5	Outcomes of the remplissage procedure and its effects on return to sports average 5‐year follow‐up^23^	Garcia, Grant H.; Wu, Hao‐Hua; Liu, Joseph N.; Huffman, G. Russell; Kelly, John D.	10.4	94
26	Arthroscopic Bankart repair with remplissage in comparison to bone block augmentation for anterior shoulder instability with bipolar bone loss: A systematic review^28^	Gouveia, Kyle; Abidi, Syed Kumail; Shamshoon, Saif; Gohal, Chetan; Madden, Kim; Degen, Ryan M.; Leroux, Timothy; Alolabi, Bashar; Khan, Moin	10	40
38	Remplissage yields similar 2‐year outcomes, fewer complications, and low recurrence compared to Latarjet across a wide range of preoperative glenoid bone loss^29^	Horinek, Jeffrey L.; Menendez, Mariano E.; Narbona, Pablo; Ladermann, Alexandre; Barth, Johannes; Denard, Patrick J.	9.3	28
24	Arthroscopic Bankart repair with remplissage versus Latarjet procedure for management of engaging Hill‐Sachs lesions with subcritical glenoid bone loss in traumatic anterior shoulder instability: A systematic review and meta‐analysis^30^	Haroun, Haitham K.; Sobhy, Mohamed H.; Abdelrahman, Amr A.	9	45
20	Arthroscopic remplissage for anterior shoulder instability: A systematic review of clinical and biomechanical studies^31^	Lazarides, Alexander L.; Duchman, Kyle R.; Ledbetter, Leila; Riboh, Jonathan C.; Garrigues, Grant E.	8.7	52
7	Hill‐Sachs remplissage, an arthroscopic solution for the engaging Hill‐Sachs lesion: 2‐to 10‐year follow‐up and incidence of recurrence^6^	Wolf, Eugene M.; Arianjam, Afshin	8	88

### Characteristics of the Top 50 Most‐Cited Remplissage Articles

**FIGURE 1:**
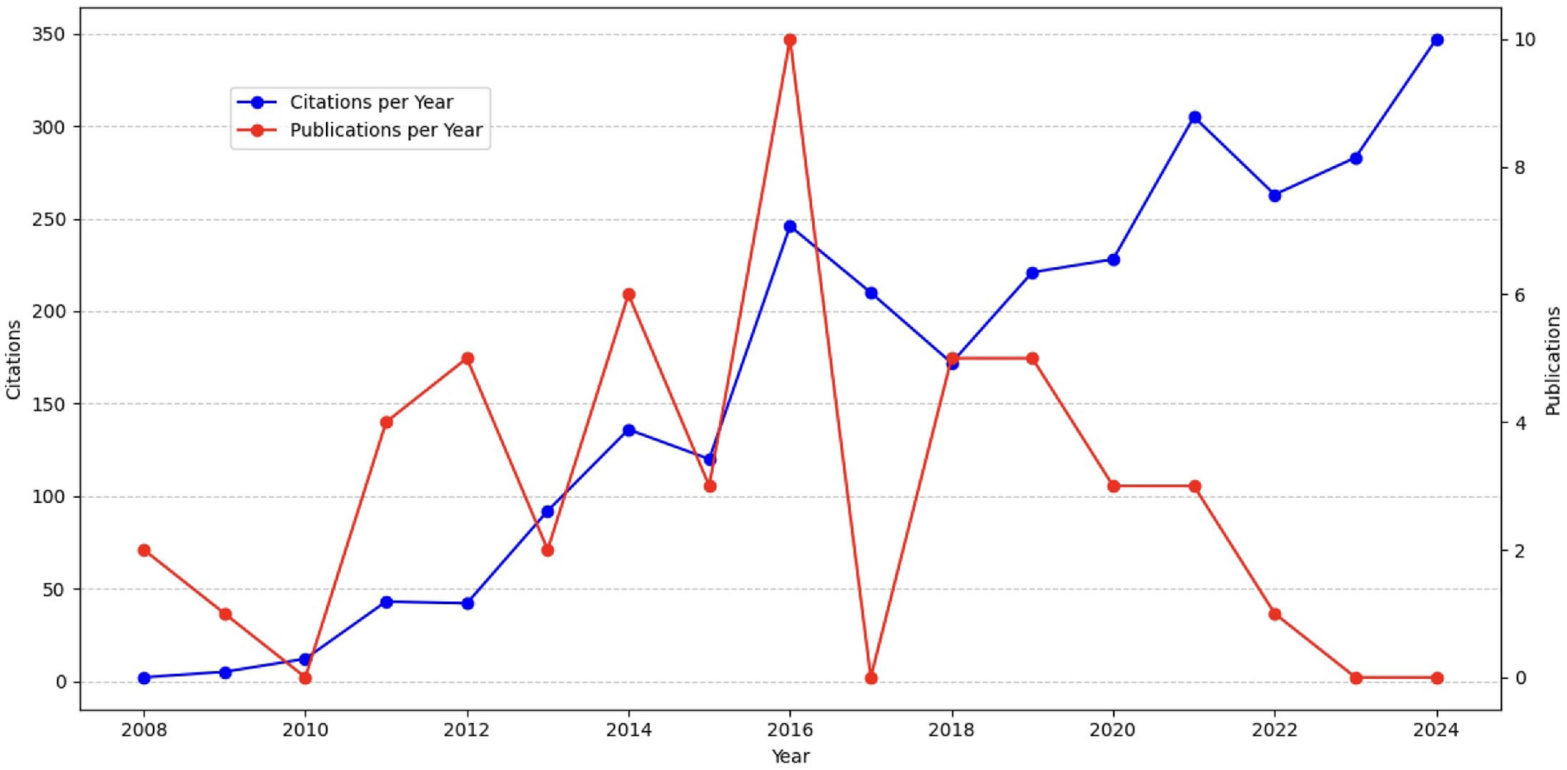
Citations and publications per year for the top 50 most‐cited remplissage articles. Citations are on an upward trajectory from 2008 to 2024, while 2016 was the year with the most articles in the top 50 most cited.

**FIGURE 2:**
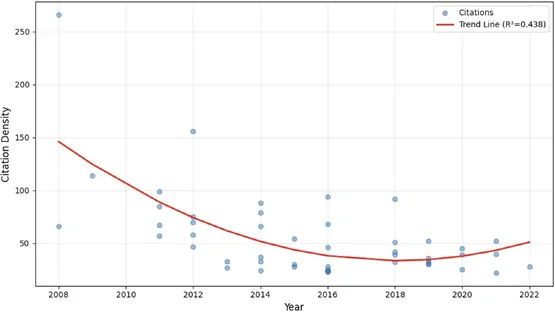
Citation density trend for the top 50 most‐cited remplissage articles. Earlier remplissage articles saw a greater number of citations than later articles, reaching a minimum in 2018 at which point an upward trend is observed.

Of the articles that were within the top 50 most‐cited articles on remplissage, the year with the greatest number of article publications from that list occurred in 2016 (n = 10, Figure 1). In terms of citations for remplissage, there has been a general trend of increasing citations from 2008 to 2024 (Figure 1). Of the top 50 most‐cited remplissage articles from the Web of Science databases, the earliest was Purchase et al. in 2008 while the latest was Hurley et al. in 2022. Citation density showed a decline in citation density from 2008 to 2018, with a slight uptrend in articles published after 2018 (Figure 2).

**FIGURE 3:**
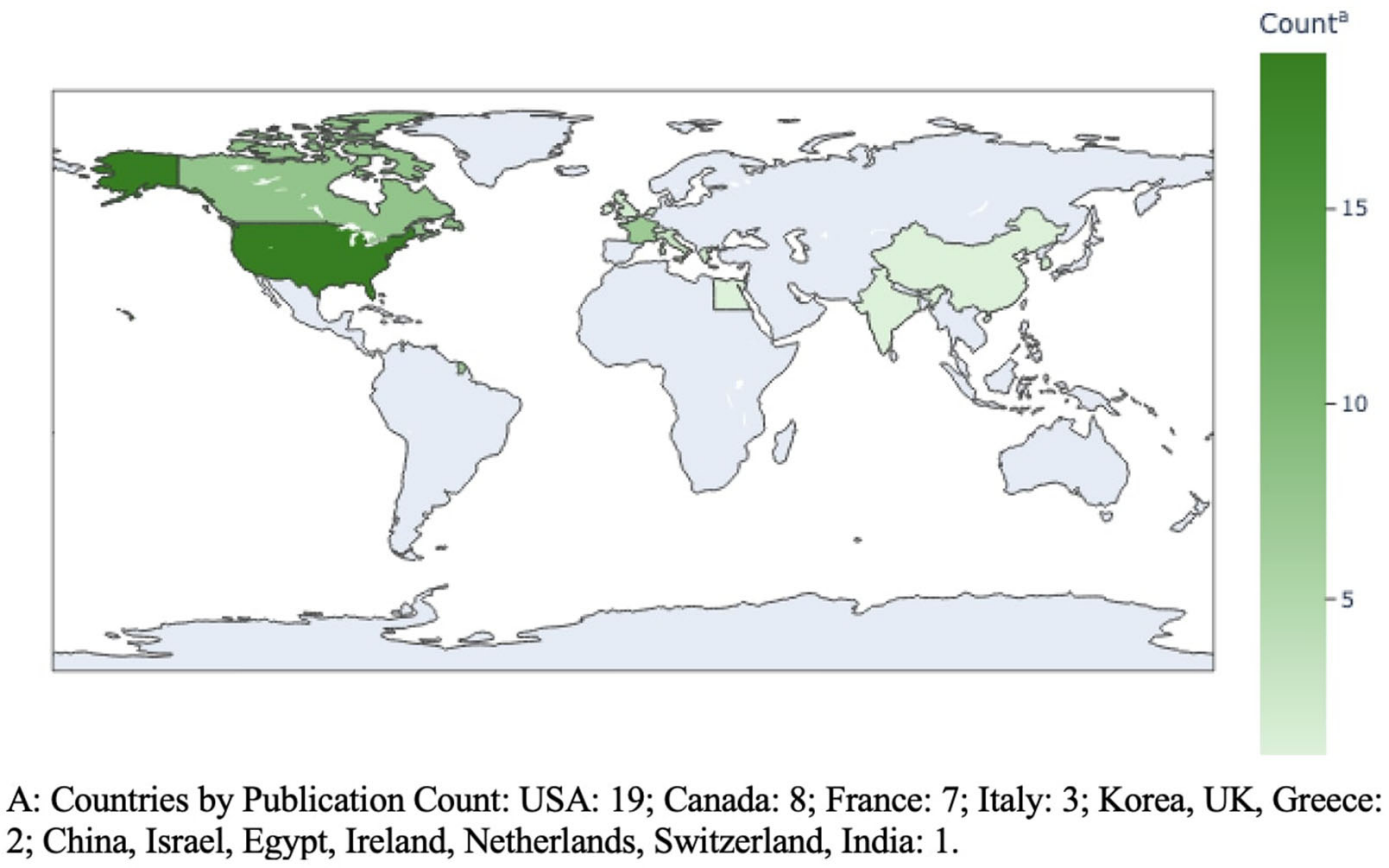
First author country distribution for top 50 most‐cited remplissage articles. USA: 19; Canada: 8; France: 7; Italy 3; Korea, UK, Greece: 2; China, Israel, Egypt, Ireland, Netherlands, Switzerland, India: 1.

A total of 15 journals made up the most‐cited remplissage articles. The most‐cited journals were *Arthroscopy* (citations = 880), *American Journal of Sports Medicine* (citations = 546), *Journal of Shoulder and Elbow Surgery* (citations = 340), and *The* *Journal of Bone and Joint Surgery – American Volume* (citations = 305). A total breakdown for each journal can be found in Table 3. When adjusted for level of evidence and study design, there was a 1.3 increase in number of CPY for every 1 unit increase in journal impact factor (95% CI: 0.28‐2.38; *P* = .02). Sensitivity analysis, excluding four outliers, yielded consistent results with no change in statistical significance (*P* = .02). The institutions that were most commonly associated with the top 50 articles were University of Pennsylvania (8%), followed by San Antonio Orthopaedic Group, University of Western Ontario, and Midwest Orthopedics at Rush all at 6% of all top‐cited remplissage articles (Table 4). Dr. Grant Garcia was the most prolific author with 5 publications in the top 50 remplissage articles, also having the most citations at 287. Second was Dr. John D. Kelly IV at 4 publications and 236 citations. Further breakdown for the top ten prolific authors can be found in Table S1. There were six senior authors who had at least two top‐cited remplissage articles. Of them, only Dr. George S. Athwal (n = 4, 8%) and Dr. John D. Kelly IV (n = 3, 6%) had more than two articles as senior author (Table S2). The majority of the top 50 remplissage articles were from the United States (n = 19, 38%), while Canada (n = 8, 16%) and France (n = 7, 14%) had the second and third most‐cited remplissage articles respectively (Figure 3).

**TABLE 3:** Journals of the Top 50 Most‐Cited Remplissage Articles

Journal	Total Citations	Count	Citations Per Year	Impact Factor (JCR 2023)
*Arthroscopy*	880	14	92.4	4.4
*American Journal of Sports Medicine*	546	7	51.5	4.2
*Journal of Shoulder and Elbow Surgery*	340	7	50	2.9
*The Journal of Bone and Joint Surgery—American Volume*	305	3	24.6	4.4
*Knee Surgery Sports Traumatology Arthroscopy*	222	7	26.4	3.3
*Orthopaedics & Traumatology: Surgery & Research*	81	2	11.6	2.3
*Orthopaedics*	66	1	3.9	1.1
*Journal of Orthopaedic Surgery and Research*	57	1	4.1	2.8
*Arthroscopy Techniques*	53	2	5.6	2.4
*Clinical Orthopaedics and Related Research*	37	1	3.4	4.4
*Shoulder & Elbow*	33	1	5.5	1.5
*Orthopaedic Journal of Sports Medicine*	32	1	4.6	2.4
*HSS Journal*	28	1	2.8	1.6
*Clinics in Orthopedic Surgery*	25	1	2.8	1.9
*Bone & Joint Journal*	22	1	5.5	4.9

JCR, journal citation reports.

**TABLE 4:** Institutions of the Top 50 Most‐Cited Remplissage Articles

Final Institutions	Publications	Total Citations	Rank List
University of Pennsylvania	4	236	5, 13, 22, 40
San Antonio Orthopaedic Group	3	188	3, 23, 39
University of Western Ontario	3	161	11, 16, 33
Midwest Orthopedics at Rush	3	131	20, 21, 38
Campus Bio‐medico University	2	141	10, 14
Hospital for Special Surgery	2	122	5, 40
Southern California Permanente Medical Group	2	120	6, 40

**FIGURE 4:**
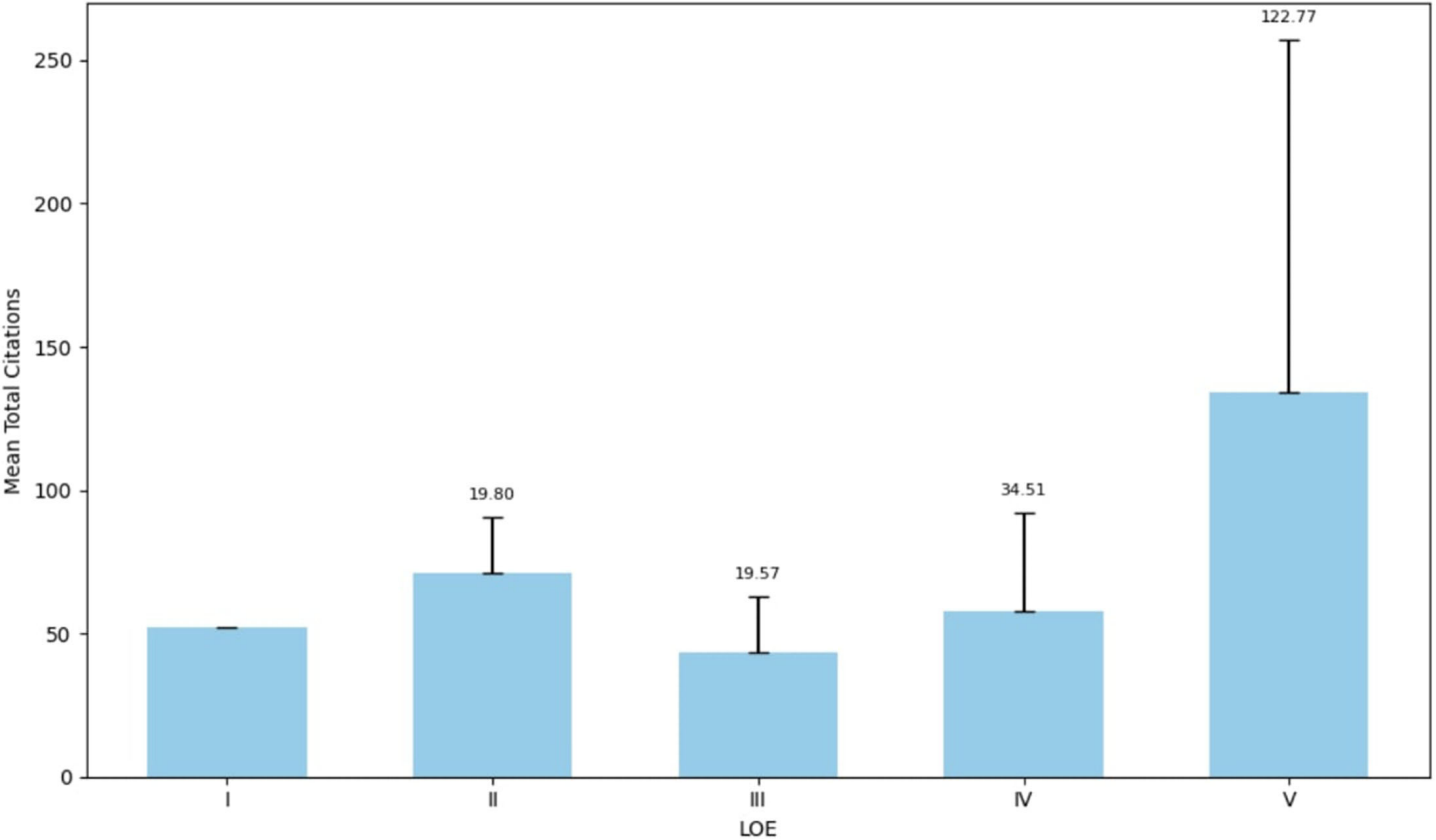
Citations by level of evidence for top 50 most‐cited remplissage articles. A majority of articles were Level IV evidence (n = 19, 58 ± 35 citations), followed by Level III evidence (n = 16, 43 ± 20 citations), Level V evidence (n = 3, 134 ± 123 citations), Level II evidence (n = 2, 71 ± 20 citations), and a single Level I evidence study (52 citations).

A total of 41 clinical studies were found in the most‐cited remplissage articles. Of these, most were Level IV evidence (n = 19, 58 ± 35 citations), followed by Level III evidence (n = 16, 43 ± 20 citations), Level V evidence (n = 3, 134 ± 123 citations), and Level II (n = 2, 71 ± 20 citations) with a single Level I study (52 citations) (Figure 4). Spearman coefficient analysis showed no association between the level of evidence and number of citations (coefficient = 0.10, *P* = .52). Of the top 50 cited remplissage articles, most were retrospective cohort studies (n = 14), followed by systematic reviews (n = 11), biomechanical studies (n = 9), retrospective case series (n = 7), technical notes (n = 3), 2 case reports, and 1 of the following (randomized control trial, case control, prospective case series, and prospective cohort) (Table S3).

**FIGURE 5:**
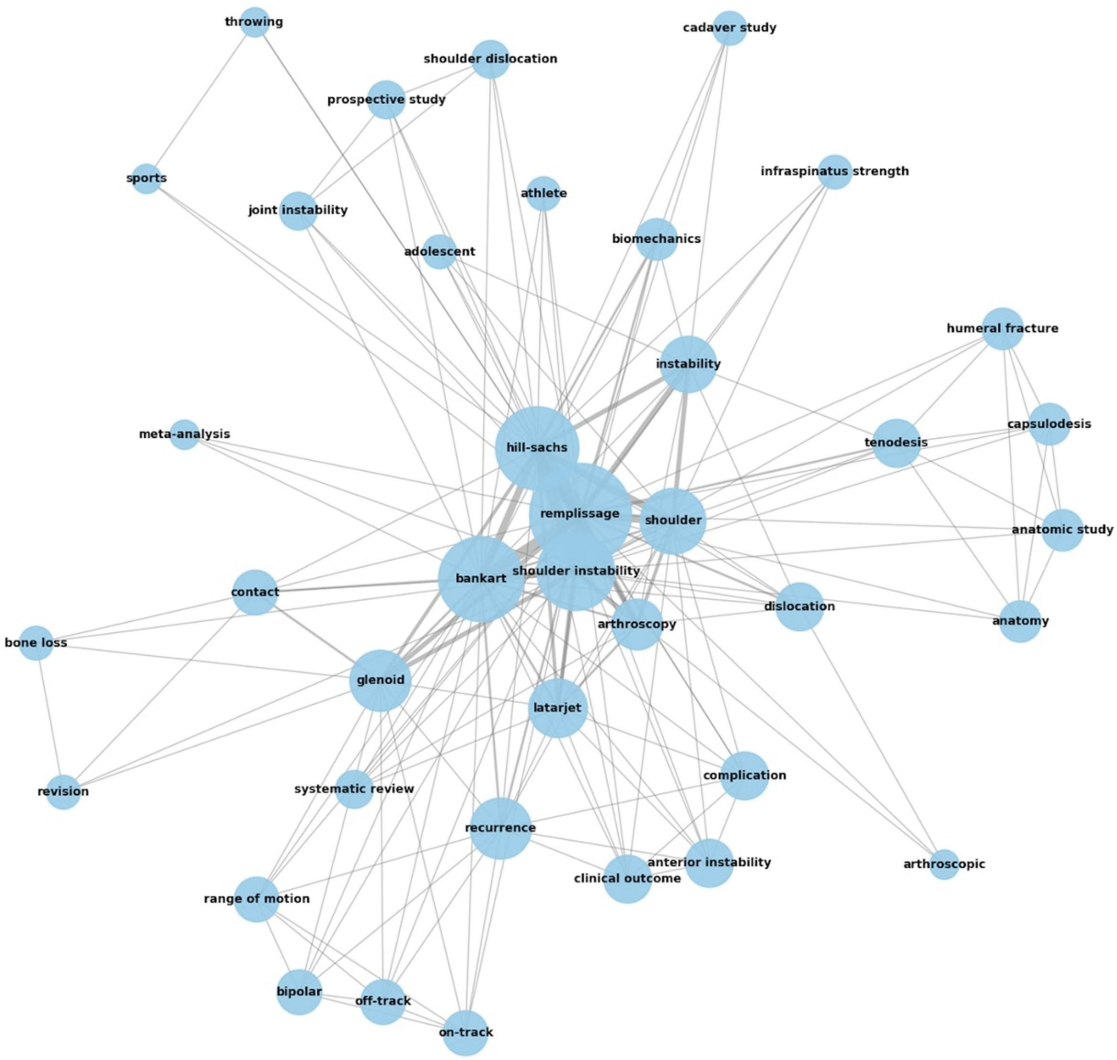
Network plot of keywords for top 50 most‐cited remplissage articles. Network analysis showed high degree of centrality for *remplissage*, *shoulder*, *shoulder instability*, *Bankart*, *arthroscopy*, and *Hill‐Sachs*. Moderate centrality was found for *Latarjet*, *glenoid*, *tenodesis*, *biomechanics*, *prospective studies*, and *shoulder dislocation*.

Keywords were found for 22 articles in the top 50 most‐cited remplissage articles. Network analysis of these keywords showed a high degree of centrality for *remplissage*, *shoulder*, *shoulder instability*, *bankart*, *arthroscopy*, and *hill‐sachs* (Figure 5).

## DISCUSSION

In our bibliometric analysis, we found that arthroscopic remplissage technique has gained steady recognition over time, as reflected by increasing citations in the literature and its growing adoption as an adjunct to ABR in patients with Hill‐Sachs lesions. While most studies within the top 50 cited articles were published in leading orthopaedic journals, the current study found no significant link between the level of evidence and citations, suggesting that remplissage consideration by surgeons might be influenced more by practical applicability than journal prestige. Conversely, the journal impact factor played a role in the number of CPY when adjusted for level of evidence and study design.

The remplissage technique is a widely accepted adjunct to the ABR in patients with a Hill‐Sachs lesion. The emerging clinical evidence has suggested reduced rates of recurrent instability in patients with Hill‐Sachs lesions—whether on‐track or off‐track—and/or with glenoid bone loss (less than the critical value) when performed with ABRs.^15^, ^27^ While concerns have been raised about potential postoperative external rotation limitations and inconsistent long‐term functional outcomes including reduced post‐operative range of motion, recent studies have provided a more nuanced perspective.

The current study highlights the growing recognition of the importance of arthroscopic remplissage procedure for the treatment of anterior glenohumeral instability, particularly in cases of recurrent instability, Hill‐Sachs lesions, off‐track lesions, and contact athletes/collision sports. The abstract that described the technique and concepts of remplissage is not indexed in common searchable databases including Pubmed, MEDLINE, or Web of Science.^7^ Hence, the earliest publication indexed on remplissage we located in our search was in 2008 by Purchase et al. The study by Purchase et al. remains the most‐cited remplissage article with 266 citations at the time of this analysis, which underscores its foundational role in defining the technique and establishing its clinical utility. Boileau et al.'s and Koo et al.'s articles, ranked second (n = 156) and third (n = 114) in citations, respectively.^9^, ^21^ These articles established the clinical evidence to further support the use of remplissage as well as a modification of the remplissage technique to include the double‐pulley technique, which is commonly employed today. In the study by Boileau et al., the authors showed that combining ABR with Hill‐Sachs remplissage effectively stabilizes the shoulder in 47 patients with large, engaging Hill‐Sachs lesions and no significant glenoid bone loss. Importantly, 100% of cases showed successful healing of the posterior capsule and infraspinatus tendon into the humeral defect, confirming the efficacy of the technique. The study reported an average external rotation loss of approximately 10°, highlighting a trade‐off between enhanced stability and slight range‐of‐motion limitations.

Koo et al. introduced the double‐pulley concept with remplissage surgery, which is now often employed as a fixation strategy. In the original remplissage technique, suture anchors were placed into the Hill‐Sachs defect from the subacromial space, with sutures passed through the posterior capsule and infraspinatus tendon before being tied within the joint. While this effectively converted the intra‐articular defect into an extra‐articular one, it could be technically demanding, including difficulty with visualization during knot tying and anchor placement.^32^ In contrast, Koo et al.'s double‐pulley technique modified the fixation approach by placing two transtendon suture anchors into the Hill‐Sachs lesion where the sutures are passed through the infraspinatus tendon and secured using a double‐pulley configuration. A key innovation in this method was moving the knot‐tying process from inside the joint to the subacromial space. Potential advantages with this approach include ease of visualization, more uniform tension, and a wider mattress‐like fixation, which provides a larger fixation footprint by ensuring broader and more stable contact between the infraspinatus tendon and the Hill‐Sachs defect.

Since the publication of Koo's initial article, several variations of the remplissage surgery have emerged making it more accessible as an adjunct technique in anterior glenohumeral instability surgery. Knotless techniques developed by Ratner et al.^33^ and Woodall et al.^34^ further expanded the fixation footprint, reduced the complexity of the procedure, and minimized the number of required portals. Additionally, the description of the double‐pulley remplissage in the beach‐chair position have been shown to potentially have some advantages including shifting the humeral head posteriorly to allow for easier access to the anterior glenoid for Bankart repair. It also allows for the standardization of patient positioning for both arthroscopic and open shoulder instability management.^35^, ^36^ These innovations simplify instrumentation and reduce operative time, thereby broadening the feasibility and adoption of remplissage across various surgical settings.

Interestingly, there was a general decrease in citation density (measuring the citations of articles in the year they were published) until 2018, followed by a precipitous increase. This may be due in part to earlier articles that were published after Wolf et al. and Purchase et al. being mostly short‐term outcome articles and biomechanical studies. As the initial short term outcomes data emerged on remplissage, the more recent publications have focused on long‐term outcomes of the remplissage technique, along with comparative studies of remplissage with Bankart repair versus isolated Bankart repair, or comparisons between Bankart repair with remplissage and the Latarjet procedure, which is performed as an alternative to Bankart repair.^27^, ^29^, ^37^, ^38^, ^39^, ^40^ The natural evolution of the clinical literature may therefore explain the variation in citation density surrounding remplissage, consistent with previous orthopaedic bibliometric studies.^41^, ^42^

In terms of the most‐cited journals on remplissage, our study identified the top journals as *Arthroscopy*, *American Journal of Sports Medicine*, *Journal of Shoulder and Elbow Surgery*, and *Knee Surgery Sports Traumatology Arthroscopy*. This is consistent with similar bibliometric studies evaluating bibliometric trends for shoulder arthroplasty, shoulder instability, and reverse total shoulder arthroplasty.^16^, ^17^, ^43^ When adjusted for level of evidence and study design, we found a significant increase of 1.3 more CPY for a single unit increase in journal impact factor (*P* = .02), showcasing the influence of a journal and its popularity on how often an article gets cited and read in the community.

The author (first or coauthor) that contributed to the most number of the top 50 publications and is the most‐cited on remplissage was Dr. Grant Garcia at five publications (rank: 5, 13, 21, 22, 39) and 287 citations, respectively.^12^, ^23^, ^44^, ^45^, ^46^ Second was Dr. John D. Kelly, IV with four publications (rank: 5, 13, 22, 39) and 236 citations.^12^, ^23^, ^45^, ^46^ The University of Pennsylvania was also the most‐cited institution. Dr. Grant Garcia and Dr. John D. Kelly, IV shared four publications that were a top 50 cited remplissage article. In a similar vein, Dr. George S. Athwal, Dr. Joshua W. Giles, and Dr. James A. Johnson were the next most common authors who were all on the same four publications (rank: 11, 16, 29, 33) with a total of 198 citations.^37^, ^38^, ^47^, ^48^ The majority of the study on remplissage were from the United States (n = 19, 38%). Canada (n = 8) and France (n = 7) were the second and third most countries respectively. Only a small majority can be seen with the United States as the most‐cited country, no substantial author or institution pattern was seen.

Most articles on the use of remplissage were Level III or IV evidence. While it would seem logical that higher level of evidence would be cited more frequently, our analysis did not find this correlation within the top 50 remplissage articles (*P* = .52). This could be explained as articles examining the outcomes of remplissage were preferred for citations, along with being easier to gather data and published compared with prospective and randomized controlled trials. There was only 1 randomized controlled trial in the top 50 remplissage articles: MacDonald et al.^27^ (2021) that compared ABR with and without remplissage in 108 patients (54 with remplissage, 54 without remplissage) in patients with less than 15% anterior glenoid bone loss. Results at 2 years follow‐up found significantly greater risk of recurrent instability in patients who did not have a remplissage performed. Remplissage was found to have significantly lower rates of redislocation in high‐risk patients with Hill‐Sachs lesion ≥20 mm and/or ≥15% in size. The author, however, reported no difference in patient reported outcomes, complications, or shoulder function if either procedure was successful in treating the instability. While there is considerable heterogeneity in article types within the literature, our analysis was designed to capture the entirety of the published remplissage experience, including technical notes, case series, and higher‐level studies with clinical outcomes.

Keywords were found in 22 of the top 50 cited remplissage articles. Network analysis showed high degree of centrality for *remplissage*, *shoulder*, *shoulder instability*, *Bankart*, *arthroscopy*, and *Hill‐Sachs*. Moderate centrality was found for *Latarjet*, showcasing a moderate proportion of studies comparing the two techniques on top of Bankart procedures. Moderate centrality was also found for keywords such as *glenoid*, *tenodesis*, *biomechanics*, *prospective studies*, and *shoulder dislocation*.

### Limitations

Like all studies, this bibliometric analysis has its limitations. One limitation is the time dependent nature of this analysis, as articles require time to accumulate citations while subsequent articles are conducted and published. We used CPY as a secondary ranking metric after total citations, so when two articles had the same citation count, the one with a higher citation rate was ranked higher. This can be seen in effect with the rank 19 and rank 20 articles, which both have 52 citations; however, MacDonald et al.^27^ had a CPY of 13, while Lazarides et al.^31^ had a CPY of 8.7 (Supporting Information 1). This significantly changes the rankings of the top articles compared with using total citations as the ranking metric (Spearman coefficient: 0.69, *P* < .01). Additionally, the outcome measure for the multivariable linear regression on journal impact factor used CPY over total citations. Another potential limitation of the current study is the limitation surrounding a single database. While multiple databases (Scopus, Google Scholar, and Medline) all have their own form of citation record keeping, there will be discrepancies between them due to which resources and journals are kept in these databases. We had to choose a single database for quantifying citation numbers. While considering other databases, Web of Science was selected due to their comprehensive breathe of articles, and the usage of various databases alongside their core collection. Furthermore, while citation counts provide an indication of impact, they may not completely reflect the methodological quality or clinical relevance of the studies. Another variable to consider around the use of citations is the potential for authors to self‐cite and the potential for a “snowball effect,” where frequently cited articles attract more references over time, leading to the probability of self‐selection bias. There is also the potential for language barriers and regional biases towards to non‐English articles that could lead to the potential for underrepresenting the global understanding and literature of remplissage at large. However, this was mitigated by using all the databases under Web of Science—including the Chinese Science Citation Database, Russian Science Citation Index, and Arabic Citation Index.

## CONCLUSIONS

No relationship was found between the level of evidence and number of citations of arthroscopic Remplissage articles; however, journal impact factor was associated with citation frequency when adjusted for study design and level of evidence.

## DISCLOSURES

The authors (K.A.H., R.L.P., R.M., X.L.) declare the following financial interests/personal relationships which may be considered as potential competing interests: K.A.H. reports a relationship with Linkbio Corp that includes consulting or advisory. R.L.P. reports a relationship with Arthrex that includes consulting or advisory and reports a relationship with Vericel Corporation that includes consulting or advisory. R.M. reports a relationship with Johnson & Johnson MedTech that includes consulting or advisory. X.L. reports a relationship with FH ORTHO SAS that includes consulting or advisory. The other authors (B.S.T., R.C., A.C., J.D.K.) declare that they have no known competing financial interests or personal relationships that could have appeared to influence the work reported in this article.

## Supporting information

Supplementary Material
