# Supplementary material for: Characteristics of the Top 50 Most‐Cited Arthroscopic Remplissage Articles Reveal Growing Recognition But Persistent Knowledge Gaps
Source: Arthrosc Sports Med Rehabil. 2026 Sep 25;8(5):e70001. doi: 10.1002/ars2.70001 (PMC13615370; doi:10.1002/ars2.70001)
Supplement: Supplementary file 1 [file ARS2-8-e70001-s001.pdf]

Supporting Information

Appendix S1: Top 50 Most-Cited Articles on Remplissage

Rank	Title	Authors	Country	Total Citations	CPY
1	Hill-Sachs Remplissage:: An arthroscopic solution for the engaging Hill-Sachs lesion[8]	Purchase, Robert J.; Wolf, Eugene M.; Hobgood, E. Rhett; Pollock, Michael E.; Smalley, Chad C.	USA	266	15.6
2	Anatomical and Functional Results After Arthroscopic Hill-Sachs Remplissage[9]	Boileau, Pascal; O'Shea, Kieran; Vargas, Pablo; Pinedo, Miguel; Old, Jason; Zumstein, Matthias	France	156	12
3	Arthroscopic Double-Pulley Remplissage Technique for Engaging Hill-Sachs Lesions in Anterior Shoulder Instability Repairs[21]	Koo, Samuel S.; Burkhart, Stephen S.; Ochoa, Eloy	USA	114	7.1
4	throscopic Bankart Repair Combined With Remplissage Technique for the Treatment of Anterior Shoulder Instability With Engaging Hill-Sachs Lesion A Report of 49 Cases With a Minimum 2-Year Follow-up[22]	Zhu, Yi-Ming; Lu, Yi; Zhang, Jin; Shen, Jie-Wei; Jiang, Chun-Yan	China	99	7.1
5	Outcomes of the Remplissage Procedure and Its Effects on Return to Sports Average 5-Year	Garcia, Grant H.; Wu, Hao-Hua; Liu, Joseph N.; Huffman, G. Russell;	USA	94	10.4

	Follow-up[23]	Kelly, John D.			
6	Remplissage Versus Modified Latarjet for Off-Track Hill-Sachs Lesions With Subcritical Glenoid Bone Loss[24]	Yang, Justin Shu; Mehran, Nima; Mazzocca, Augustus D.; Pearl, Michael L.; Chen, Vincent W.; Arciero, Robert A.	USA	92	13.1
7	Hill-Sachs remplissage, an arthroscopic solution for the engaging Hill-Sachs lesion: 2-to 10-year follow-up and incidence of recurrence[6]	Wolf, Eugene M.; Arianjam, Afshin	USA	88	8
8	A Prospective, Comparative, Radiological, and Clinical Study of the Influence of the Remplissage Procedure on Shoulder Range of Motion After Stabilization by Arthroscopic Bankart Repair[11]	Nourissat, Geoffroy; Kilinc, Alexandre Sahin; Werther, Jean Roger; Doursounian, Levon	France	85	6.1
9	Arthroscopic Hill-Sachs Remplissage A Systematic Review[25]	Buza, John A., III; Iyengar, Jaicharan J.; Anakwenze, Oke A.; Ahmad, Christopher S.; Levine, William N.	USA	79	7.2
10	Remplissage Repair-New Frontiers in the Prevention of Recurrent Shoulder Instability A 2-Year Follow-up Comparative Study[26]	Franceschi, Francesco; Papalia, Rocco; Rizzello, Giacomo; Franceschetti, Edoardo; Del Buono, Angelo; Panasci, Manlio; Maffulli, Nicola; Denaro, Vincenzo	Italy	75	5.8
11	The Effect of the Remplissage Procedure on Shoulder Stability and Range of Motion[48]	Elkinson, Ilia; Giles, Joshua W.; Faber, Kenneth J.; Boons, Harm W.;	Canada	70	5.4

		Ferreira, Louis M.; Johnson, James A.; Athwal, George S.			
12	Management of an engaging Hill-Sachs lesion: arthroscopic remplissage with Bankart repair versus Latarjet procedure[49]	Cho, Nam Su; Yoo, Jae Hyun; Rhee, Yong Girl	Korea	68	7.6
13	Arthroscopic Remplissage With Bankart Repair for the Treatment of Glenohumeral Instability With Hill-Sachs Defects	Park, Min Jung; Tjoumakaris, Fotios P.; Garcia, Grant; Patel, Amit; Kelly, John D.	USA	67	4.8
14	Remplissage, Humeral Osteochondral Grafts, Weber Osteotomy, and Shoulder Arthroplasty for the Management of Humeral Bone Defects in Shoulder Instability: Systematic Review and Quantitative Synthesis of the Literature	Longo, Umile Giuseppe; Loppini, Mattia; Rizzello, Giacomo; Ciuffreda, Mauro; Berton, Alessandra; Maffulli, Nicola; Denaro, Vincenzo	Italy	66	6
15	Decreased range of motion following arthroscopic remplissage	Deutsch, Allen A.; Kroll, Derek G.	USA	66	3.9
16	Moderate to large engaging Hill-Sachs defects: an in vitro biomechanical comparison of the remplissage procedure, allograft humeral head reconstruction, and partial resurfacing arthroplasty	Giles, Joshua W.; Elkinson, Ilia; Ferreira, Louis M.; Faber, Kenneth J.; Boons, Harm; Litchfield, Robert; Johnson, James A.; Athwal, George S.	Canada	58	4.5
17	Outcomes of arthroscopic Remplissage: capsulotenodesis of the engaging large Hill-Sachs lesion	Haviv, Barak; Mayo, Lee; Biggs, Daniel	Israel	57	4.1

18	Outcomes of Arthroscopic Hill-Sachs Remplissage and Anterior Bankart Repair A Retrospective Controlled Study Including Ultrasound Evaluation of Posterior Capsulotenodesis and Infraspinatus Strength Assessment	Merolla, Giovanni; Paladini, Paolo; Di Napoli, Giuseppe; Campi, Fabrizio; Porcellini, Giuseppe	Italy	54	5.4
19	Arthroscopic Bankart repair with and without arthroscopic infraspinatus remplissage in anterior shoulder instability with a Hill-Sachs defect: a randomized controlled trial	MacDonald, Peter; McRae, Sheila; Old, Jason; Marsh, Jonathan; Dubberley, Jamie; Stranges, Greg; Koenig, James; Leiter, Jeff; Mascarenhas, Randy; Prabhakar, Sharad; Sasyniuk, Treny; Lapner, Peter	Canada	52	13
20	Arthroscopic Remplissage for Anterior Shoulder Instability: A Systematic Review of Clinical and Biomechanical Studies	Lazarides, Alexander L.; Duchman, Kyle R.; Ledbetter, Leila; Riboh, Jonathan C.; Garrigues, Grant E.	USA	52	8.7
21	Recurrence Rate of Instability After Remplissage for Treatment of Traumatic Anterior Shoulder Instability: A Systematic Review in Treatment of Subcritical Glenoid Bone Loss	Liu, Joseph N.; Gowd, Anirudh K.; Garcia, Grant H.; Cvetanovich, Gregory L.; Cabarcas, Brandon C.; Verma, Nikhil N.	USA	51	7.3
22	The Evaluation of Arthroscopic Remplissage by High-Resolution Magnetic Resonance Imaging	Park, Min Jung; Garcia, Grant; Malhotra, Amit; Major, Nancy; Tjoumakaris, Fotios P.; Kelly, John D.	USA	47	3.6

23	Remplissage of an Off-track Hill-Sachs Lesion Is Necessary to Restore Biomechanical Glenohumeral Joint Stability in a Bipolar Bone Loss Model	Hartzler, Robert U.; Bui, Christopher N. H.; Jeong, Woong K.; Akeda, Masaki; Peterson, Alex; McGarry, Michelle; Denard, Patrick J.; Burkhart, Stephen S.; Lee, Thay Q.	USA	46	5.1
24	Arthroscopic Bankart repair with remplissage versus Latarjet procedure for management of engaging Hill-Sachs lesions with subcritical glenoid bone loss in traumatic anterior shoulder instability: a systematic review and meta-analysis	Haroun, Haitham K.; Sobhy, Mohamed H.; Abdelrahman, Amr A.	Egypt	45	9
25	Isolated arthroscopic Bankart repair vs. Bankart repair with remplissage for anterior shoulder instability with engaging Hill-Sachs lesion: A meta-analysis	Camus, Dimitri; Domos, Peter; Berard, Emilie; Toulemonde, Julien; Mansat, Pierre; Bonnevialle, Nicolas	France	42	6
26	Arthroscopic Bankart Repair With Remplissage in Comparison to Bone Block Augmentation for Anterior Shoulder Instability With Bipolar Bone Loss: A Systematic Review	Gouveia, Kyle; Abidi, Syed Kumail; Shamshoon, Saif; Gohal, Chetan; Madden, Kim; Degen, Ryan M.; Leroux, Timothy; Alolabi, Bashar; Khan, Moin	Canada	40	10
27	Remplissage for anterior shoulder instability with Hill-Sachs lesions: a systematic review and meta-analysis	Hurley, Eoghan T.; Toale, James P.; Davey, Martin S.; Colasanti, Christopher A.; Pauzenberger, Leo; Strauss, Eric J.; Mullett, Hannan	Ireland	39	7.8

28	Chronic anterior shoulder instability with significant Hill-Sachs lesion: Arthroscopic Bankart with remplissage versus open Latarjet procedure	Bah, A.; Lateur, G. M.; Kouevidjin, B. T.; Bassinga, J. Y. S.; Issa, M.; Jaafar, A.; Beaudouin, E.	France	39	5.6
29	Remplissage Versus Latarjet for Engaging Hill-Sachs Defects Without Substantial Glenoid Bone Loss: A Biomechanical Comparison	Degen, Ryan M.; Giles, Joshua W.; Johnson, James A.; Athwal, George S.	Canada	37	3.4
30	Remplissage With Bankart Repair in Anterior Shoulder Instability: A Systematic Review of the Clinical and Cadaveric Literature	Alkaduhimi, Hassanin; Verweij, Lukas P. E.; Willigenburg, Nienke W.; van Deurzen, Derek F. P.; van den Bekerom, Michel P. J.	Netherlands	36	6
31	Arthroscopic Bankart repair with remplissage for non-engaging Hill-Sachs lesion in professional collision athletes.	Domos, Peter; Ascione, Francesco; Wallace, Andrew L	UK	33	5.5
32	Primary Versus Revision Arthroscopic Reconstruction With Remplissage for Shoulder Instability With Moderate Bone Loss	McCabe, Michael P.; Weinberg, Douglas; Field, Larry D.; O'Brien, Michael J.; Hobgood, E. Rhett; Savoie, Felix H., III	USA	33	3
33	The shoulder remplissage procedure for Hill-Sachs defects: does technique matter?	Elkinson, Iia; Giles, Joshua W.; Boons, Harm W.; Faber, Kenneth J.; Ferreira, Louis M.; Johnson, James A.; Athwal, George S.	Canada	33	2.8
34	Arthroscopic Bankart Repairs With and Without	Hughes, Jessica L.; Bastrom, Tracey;	USA	32	4.6

	Remplissage in Recurrent Adolescent Anterior Shoulder Instability With Hill-Sachs Deformity	Pennock, Andrew T.; Edmonds, Eric W.			
35	Long-term outcome of arthroscopic remplissage in addition to the classic Bankart repair for the management of recurrent anterior shoulder instability with engaging Hill-Sachs lesions	Brilakis, Emmanouil; Avramidis, Grigoris; Malahias, Michael-Alexander; Stathellis, Apostolos; Deligeorgis, Anastasios; Chiotis, Ioannis; Mataragas, Elias; Chronopoulos, Efstathios; Antonogiannakis, Emmanouil	Greece	31	5.2
36	Impact of Remplissage on Global Shoulder Outcome: A Long-Term Comparative Study	Bastard, Claire; Herisson, Olivier; Gaillard, Julien; Nourissat, Geoffroy	France	30	5
37	Use of 3-Dimensional Printing for Preoperative Planning in the Treatment of Recurrent Anterior Shoulder Instability.	Sheth, Ujash; Theodoropoulos, John; Abouali, Jihad	Canada	30	3
38	Remplissage Yields Similar 2-Year Outcomes, Fewer Complications, and Low Recurrence Compared to Latarjet Across a Wide Range of Preoperative Glenoid Bone Loss	Horinek, Jeffrey L.; Menendez, Mariano E.; Narbona, Pablo; Ladermann, Alexandre; Barth, Johannes; Denard, Patrick J.	USA	28	9.3
39	Is arthroscopic remplissage a tenodesis or capsulomyodesis? An anatomic study	Laedermann, Alexandre; Arrigoni, Paolo; Barth, Johannes; Narbona, Pablo; Hanypsiak, Bryan; Burkhart, Stephen S.; Denard, Patrick J.	Switzerland	28	3.1
40	Large Hill-Sachs Lesion: a Comparative Study of	Garcia, Grant H; Park, Min Jung;	USA	28	2.8

	Patients Treated with Arthroscopic Bankart Repair with or without Remplissage.	Zhang, Clare; Kelly, John D 4th; Huffman, G Russell			
41	Combined Arthroscopic Bankart Repair and Remplissage for Recurrent Shoulder Instability	Leroux, Timothy; Bhatti, Arman; Khoshbin, Amir; Wasserstein, David; Henry, Patrick; Marks, Paul; Takhar, Kirat; Veillette, Christian; Theodoropolous, John; Chahal, Jaskarndip	Canada	27	2.2
42	A retrospective cohort analysis of arthroscopic Bankart repair with or without remplissage in patients with off-track Hill-Sachs lesion evaluated for functional outcomes, recurrent instability, and range of motion	Pandey, Vivek; Gangadharaiah, Lohith; Madi, Sandesh; Acharya, Kiran; Nayak, Shalini; Karegowda, Lakshmikanth H.; Willems, W. Jaap	India	25	5
43	The Influence of Arthroscopic Remplissage for Engaging Hill-Sachs Lesions Combined with Bankart Repair on Redislocation and Shoulder Function Compared with Bankart Repair Alone.	Ko, Sang-Hun; Cha, Jae-Ryong; Lee, Chae-Chil; Hwang, Il-Yeong; Choe, Chang-Gyu; Kim, Min-Seok	Korea	25	2.8
44	The biomechanical effect of shoulder remplissage combined with Bankart repair for the treatment of engaging Hill-Sachs lesions	Argintar, Evan; Heckmann, Nathanael; Wang, Lawrence; Tibone, James E.; Lee, Thay Q.	USA	24	2.7
45	Arthroscopic Remplissage for shoulder instability: a systematic review	Rashid, Mustafa S.; Crichton, James; Butt, Usman; Akimau, Pavel I.; Charalambous, Charalambos P.	UK	24	2.7

46	Bankart repair versus Bankart repair plus remplissage: an in vitro biomechanical comparative study	Grimberg, Jean; Diop, Amadou; Ghosn, Rosny Bou; Lanari, Dimitri; Canonne, Adrien; Maurel, Nathalie	France	24	2.7
47	The Effect of the Remplissage Procedure on Shoulder Range of Motion: A Cadaveric Study	Omi, Rei; Hooke, Alexander W.; Zhao, Kristin D.; Matsuhashi, Tomoya; Goto, Akira; Yamamoto, Nobuyuki; Sperling, John W.; Steinmann, Scott P.; Itoi, Eiji; An, Kai-Nan	USA	24	2.2
48	Midterm outcomes of arthroscopic remplissage for the management of recurrent anterior shoulder instability	Brilakis, Emmanouil; Mataragas, Elias; Deligeorgis, Anastasios; Maniatis, Vasilios; Antonogiannakis, Emmanouil	Greece	23	2.6
49	Arthroscopic Reverse Remplissage for Posterior Instability.	Lavender, Chad D; Hanzlik, Shane R; Pearson, Sara E; Caldwell, Paul E 3rd	USA	23	2.6
50	Glenoid erosion is a risk factor for recurrent instability after Hill-Sachs remplissage	Cavalier, M.; Johnston, T. R.; Tran, L.; Gauci, M-O; Boileau, P.	France	22	5.5

Table S1: Top Ten Most-Cited Authors for Remplissage Articles

Authors	Publications	Total Citations	Rank	Institution
Garcia, Grant	5	287	5, 13, 21, 22, 40	Proliance Surgeons Orthopedic Specialists of Seattle, 2409 N. 45Th Street, Seattle, WA, 98103 , USA.
Kelly, John D 4th	4	236	5, 13, 22, 40	Department of Orthopaedic Surgery, University of Pennsylvania, Philadelphia, PA, USA
Athwal, George S.	4	198	11, 16, 29, 33	Roth, McFarlane Hand and Upper Limb Centre, St Joseph's Health Care, London, Canada
Giles, Joshua W.	4	198	11, 16, 29, 33	Department of Orthopaedics, University of British Columbia, Vancouver, BC V8P 5C2, Canada
Johnson, James A.	4	198	11, 16, 29, 33	Roth, McFarlane Hand and Upper Limb Centre, St Joseph's Health Care, London, Canada
Burkhart, Stephen S.	3	188	3, 23, 39	Burkhart Research Institute for Orthopedics, San Antonio, Texas, U.S.A.
Boons, Harm	3	161	11, 16, 33	Elkerliek Ziekenhuis, Wesselmanlaan 25, 5707 HA Helmond, The Netherlands.
Elkinson, Iia	3	161	11, 16, 33	Hand and Upper Limb Centre, St. Joseph's Health Centre, The University of Western Ontario, London,

				ON, Canada.
Faber, Kenneth J.	3	161	11, 16, 33	Roth, McFarlane Hand and Upper Limb Centre, St Joseph's Health Care, London, Canada
Ferreira, Louis M.	3	161	11, 16, 33	Roth, McFarlane Hand and Upper Limb Centre, St Joseph's Health Care, London, Canada

Table S2: Senior Authors with at least Two Top 50 Most-Cited Remplissage Articles

**Senior Author**	**Publications**	**Total Citations**	**Rank List**	**Current Institution**
Athwal, George S.	4	198	11, 16, 29, 33	Roth, McFarlane Hand and Upper Limb Centre, St Joseph's Health Care, London, Canada
Kelly, John D.	3	208	5, 13, 22	Department of Orthopaedic Surgery, University of Pennsylvania, Philadelphia, PA, USA
Denaro, Vincenzo	2	141	10, 14	Department of Orthopaedic and Trauma Surgery, Campus Bio-Medico University, Rome, Italy
Lee, Thay Q.	2	70	23, 44	Orthopaedic Biomechanics Laboratory, VA Long Beach Healthcare System (09/151), 5901 East 7th. Street, Long Beach, CA, 90822, USA.
Denard, Patrick J.	2	56	38, 39	Oregon Shoulder Institute, Medford, Oregon, U.S.A.
Antonogiannakis, Emmanouil	2	54	35, 48	2nd Orthopaedic Department, Shoulder Arthroscopy and Surgery Center, IASO General Hospital, 44-46 Str. Sevastopoulou, 115 24, Athens, Greece

Table S3: Distribution of Article Types for the Top 50 Most Cited Remplissage Articles

**Study Type**	**Count**
Retrospective cohort studies	14
Systematic reviews	11
Biomechanical studies	9
Retrospective case series	7
Technical notes	3
Case reports	2
Randomized control trial	1
Case control	1
Prospective case series	1
Prospective cohort	1
